# High Ambient Temperatures Are Associated With Reduced Foraging Capacity in an Equatorial Mammal, the Banded Mongoose (*Mungos mungo*)

**DOI:** 10.1002/ece3.71872

**Published:** 2025-07-29

**Authors:** Monil Khera, Kevin Arbuckle, Onismus Bwambale, Francis Mwanguhya, Megan H. Nicholl, Michael A. Cant, Hazel J. Nichols

**Affiliations:** ^1^ Department of Biosciences Swansea University Swansea UK; ^2^ Faculty of Medicine, Health and Life Science, Swansea University Swansea UK; ^3^ Banded Mongoose Research Project, Queen Elizabeth National Park Lake Katwe Kasese District Uganda; ^4^ Centre for Ecology and Conservation, University of Exeter Cornwall UK

**Keywords:** banded mongoose, climate change, equatorial, foraging, thermoregulation

## Abstract

Due to anthropogenic climate change, there is substantial interest in how temperature variation impacts reproduction and survival in animal populations; however, the underlying mechanisms are often poorly understood. Animals often behaviourally thermoregulate under high ambient temperatures (*T*_a_s) to avoid their body temperatures rising, for instance, becoming less active and resting in shade. However, this can trade off with performing vital activities, including foraging, reproduction and social behaviours. We studied a Ugandan population of banded mongooses ( *Mungos mungo* ) to investigate how changing temperatures impact behaviour. We found that banded mongooses reduce activity under high *T*_a_s, such that foraging opportunities, in particular, are constrained. This may explain why previous studies on this species have found that offspring care is reduced under high *T*_a_s, resulting in lower pup weight and survival, as adults may struggle to meet their daily food requirements and therefore prioritise their own survival over helping to raise pups. As global temperatures continue to increase, lowland equatorial species (which are already subject to high *T*_a_s) may struggle to both behaviourally thermoregulate and maintain energy intake through foraging. Our study highlights the importance of fine‐scale quantification of behaviours in wild systems for understanding the mechanisms underlying the impact of changing environmental conditions on natural populations.

## Graphical abstract

Our study investigated behavioural thermoregulation in a population of wild banded mongooses found on the equator living under narrow thermal ranges and so potentially vulnerable to even small increases in temperature. We collected fine‐scale behavioural and environmental data and found evidence of behavioural thermoregulation, with individuals foraging less and resting more under higher ambient temperatures, leading to a bimodal activity pattern. This suggests that individuals may face a trade‐off between thermoregulation and foraging, with potential negative consequences for body condition and survival.

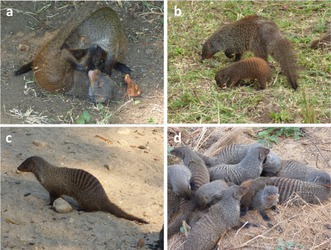

## 1 Introduction

Human‐induced climate change is leading to increased average ambient temperatures (*T*_a_s) and thermal extremes (Donat and Alexander 2012; Lee et al. 2021). This could make thermoregulation difficult for many species, which may result in harmful hyperthermia (Lovegrove et al. 2014; Conradie et al. 2020), reduced physical condition (Gardner et al. 2016; Sharpe et al. 2019), increased mortality (McKechnie and Wolf 2010; Bourne et al. 2020a, 2020b) and impaired reproduction (McNutt et al. 2019). Whilst the impact of climate change on fitness is well studied, it is not always clear what mechanisms underlie these effects (Helmuth et al. 2005). Along with morphological and physiological thermoregulatory mechanisms, animals may be able to reduce some of the costs of being exposed to high *T*_a_s by changing their behaviours, a strategy known as'behavioural thermoregulation’ (Terrien et al. 2011). Understanding the extent to which behaviours can help to mitigate against harsh conditions is important when estimating the impact of climate change on extinction risk (Enriquez‐Urzelai et al. 2020).

The most widespread form of behavioural thermoregulation is to minimise heat gain from the environment by seeking cool microsites such as shaded areas, burrows and tree canopies (Giotto et al. 2013; Lopes and Bicca‐Marques 2017). Similarly, reducing activity levels minimises metabolic heat production, which can also minimise heat gain (Cain et al. 2006). This can result in bimodal activity patterns, with many animals becoming crepuscular under high *T*_a_s; whereby activity is concentrated in the mornings and evenings, with animals primarily resting in the shade during the hotter hours of the day (Carmi‐Winkler et al. 1987; Hinsley 1994; Williams 2001). For example, high *T*_a_s have been shown to reduce foraging behaviour of the alpine ibex ( *Capra ibex* ) during the middle of the day (Aublet et al. 2009) and increase nocturnal foraging (Brivio et al. 2024).

Whilst some thermoregulatory behaviours reduce heat gain, others increase heat loss. For example, wallowing or covering the body in mud or water increases conductive and evaporative heat loss in large mammals (McKay 1973; Mota‐Rojas et al. 2021). Panting or otherwise exposing mucous membranes to the air can also be an effective way to cool through evaporative heat loss (Dawson 1982; Loughran and Wolf 2023). This strategy is most effective in low humidity because evaporative cooling requires body water vapour to move into the surrounding air across a humidity gradient, such that the rate of evaporative heat loss is directly related to the difference in humidity between the surface of the animal and the environment (Powers 1992; Gerson et al. 2014; Mitchell et al. 2018). Finally, animals may reduce physical contact with each other at high *T*_a_s, as maintaining close contact can impair cooling (Gilbert et al. 2010), although contact between individuals can potentially create shade and hence reduce exposure to solar radiation (Cain et al. 2006).

Although behavioural thermoregulation can be effective, the strategies used can often incur a fitness cost. For example, reducing activity and resting in the shade during hot periods leads to lost opportunities for foraging and may lead to reduced energy intake and lower body condition (Aublet et al. 2009). This is seen in the southern yellow‐billed hornbills ( *Tockus leucomelas* ), where thermoregulation under hot conditions via panting behaviour and selecting cooler microhabitats results in a reduction in foraging efficiency with negative consequences for body condition (Van de Ven et al. 2019). Behavioural thermoregulation may also place constraints on social behaviour; for example, a reduction in physical contact between individuals may constrain social bonding behaviours such as grooming.

Thermoregulation may pose a particular problem for species living in the tropics (Deutsch 2008; Sinervo et al. 2010; Lovegrove et al. 2014) where the *T*_a_ may be more likely to exceed their thermal prescriptive zone; hence, physiological malfunction starts to occur (Mitchell et al. 2018). Whilst endotherms can maintain fixed core temperatures under a range of ambient temperatures, this may result in significant energy costs, and so endotherms can also have narrow thermal niches (range of conditions under which they are able to survive) (Porter and Kearney 2009). Furthermore, since tropical lowland species are adapted to living under narrow *T*_a_ ranges, they may be disproportionately vulnerable to even small increases in *T*_a_ (Tewksbury et al. 2008; Bozinovic et al. 2011; Wright et al. 2009). Results by Thonis et al. (2020) however suggest that some tropical mammals, such as lesser treeshrews ( *Tupaia minor* ), may be able to better deal with high *T*_a_s than others and propose that more research is required to accurately predict how sensitive such species may be to increasing *T*_a_s. Despite this, most previous studies on the behavioural responses of mammals to climate change have focused on temperate or subtropical regions, with equatorial mammals receiving little attention (Lovegrove et al. 2014; Sheldon 2019).

Banded mongooses ( *Mungos mungo* ) present an excellent opportunity to study fine‐scale behavioural changes in response to *T*_a_. Equatorial populations of this species experience little seasonal variation in *T*_a_, but short‐term variation is considerable, allowing us to compare activity levels and behaviour under a relatively large range of *T*_a_s. Although no formal study has previously been conducted, observations over ~25 years find that banded mongooses display a distinctly bimodal activity pattern, being more active and foraging during the early morning and afternoon and resting in the shade during the middle of the day when temperatures are the highest (Rood 1975; Cant et al. 2013). Whilst this activity pattern may in part be due to individuals being satiated after their morning foraging session and therefore resting during the middle of the day, under cooler conditions, banded mongooses have been observed to forage almost continuously throughout the day, with only short rest intervals (Rood 1975). This supports our hypothesis that behavioural thermoregulation at higher temperatures could lead to a reduction in foraging behaviour.

Banded mongooses also engage in huddling behaviour, whereby they aggregate with their bodies touching each other (Gilbert et al. 2010). It has been suggested that they do this during cooler mornings to reduce heat loss (Rood 1975); however, banded mongooses also huddle during the middle of the day (pers. obs.), suggesting that it may not be associated with retaining heat. It is also possible that huddling occurs under hotter conditions as a result of individuals competing for limited cooler microsites. This is a proposed explanation for why southern pied babblers ( *Turdoides bicolor* ) are observed huddling at the base of trees at high ambient temperatures (Bourne and Soravia 2023).

Our study aims to understand how *T*_a_ influences banded mongoose behaviour. More specifically, we investigate how behaviour varies in response to changing *T*_a_s. We focus on activity levels, foraging, resting, and huddling behaviours, which may all be involved in thermoregulation and could also impact fitness. We predict that mongooses will become less active under hotter conditions, spending more time resting and less time foraging and huddling.

## 2 Materials and Methods

### 2.1 Study Site and Population

Our study was conducted in May 2023 on a wild social group of 38 banded mongooses residing in Queen Elizabeth National Park, Mweya, Uganda (0°12′S, 27° 54′ E). The group consisted of 24 males and 14 females (aged from 2.5 months to just over 7 years old) which foraged together during the day, either as one large group or sometimes splitting into two smaller subgroups. There were also 12 pups present in the group, which were too young to accompany the group on foraging trips and instead remained in the den and were ‘babysat’ by 1–5 adults. Babysitters defend the pups from potential predators and attacks from rival groups (Cant et al. 2016). Although adults forage in close proximity to each other, each individual forages independently and defends their prey from other individuals (Rood 1975; De Luca and Ginsberg 2001). Banded mongooses primarily feed on a variety of small invertebrates including millipedes, ants, beetles and termites, though they also occasionally eat small vertebrates (Rood 1975) and forage from anthropogenic sources at our study site such as waste bins. Banded mongooses appear to locate prey by smell, and since most of their prey is found in the first ~10 cm of soil, they typically use their forepaws to dig up prey (Rood 1975; Cant et al. 2013). The territory of the social group studied incorporated a village and safari lodge, from which the mongooses could sometimes forage on anthropogenic food sources; however, the vast majority of foraging time was spent foraging for natural prey. All individuals in the group could be identified by unique ‘haircuts’ (Hodge 2007; Jordan et al. 2010) and were habituated to observers at ~1 m, which allowed detailed behavioural and thermal data to be collected.

Our study site is located only ~20 km from the equator, and as a consequence, there is very little seasonal variation in *T*_a_s (monthly mean maximum daily *T*_a_ ± SD = 29.5°C ± 2.5°C). There is therefore no hotter summer or colder winter period in this study site, as would be expected in temperate regions. *T*_a_s do however vary during the course of each day, with minimum *T*_a_s typically dipping during the night (mean minimum daily *T*_a_ ± SD = 20.2°C ± 2.0°C). *T*_a_s have been increasing in Uganda by on average 0.3°C per decade since the 1960's and are predicted to continue to increase by 2°C–2.5°C by 2065–2095 (GoU 2007; GoU 2015). Rainfall is characterised by two distinct wet seasons which occur from March to May and August to December (Marshall et al. 2016). Rainfall is linked with food availability for banded mongooses. For example, millipedes, which are an important component of the banded mongoose diet, are less frequently consumed during dry months (Rood 1975). This is likely because during the dry season millipedes bury themselves up to 20 cm underground, making it challenging to dig them up (Dangerfield and Telford 1991; De Luca 1998). Supporting this link between rainfall and food availability, high rainfall is positively associated with physical condition (Nichols et al. 2012; Bell et al. 2012). Our study took place at the end of the March–May wet season when natural food availability was likely to be relatively high.

### 2.2 Data Collection

**FIGURE 1:**
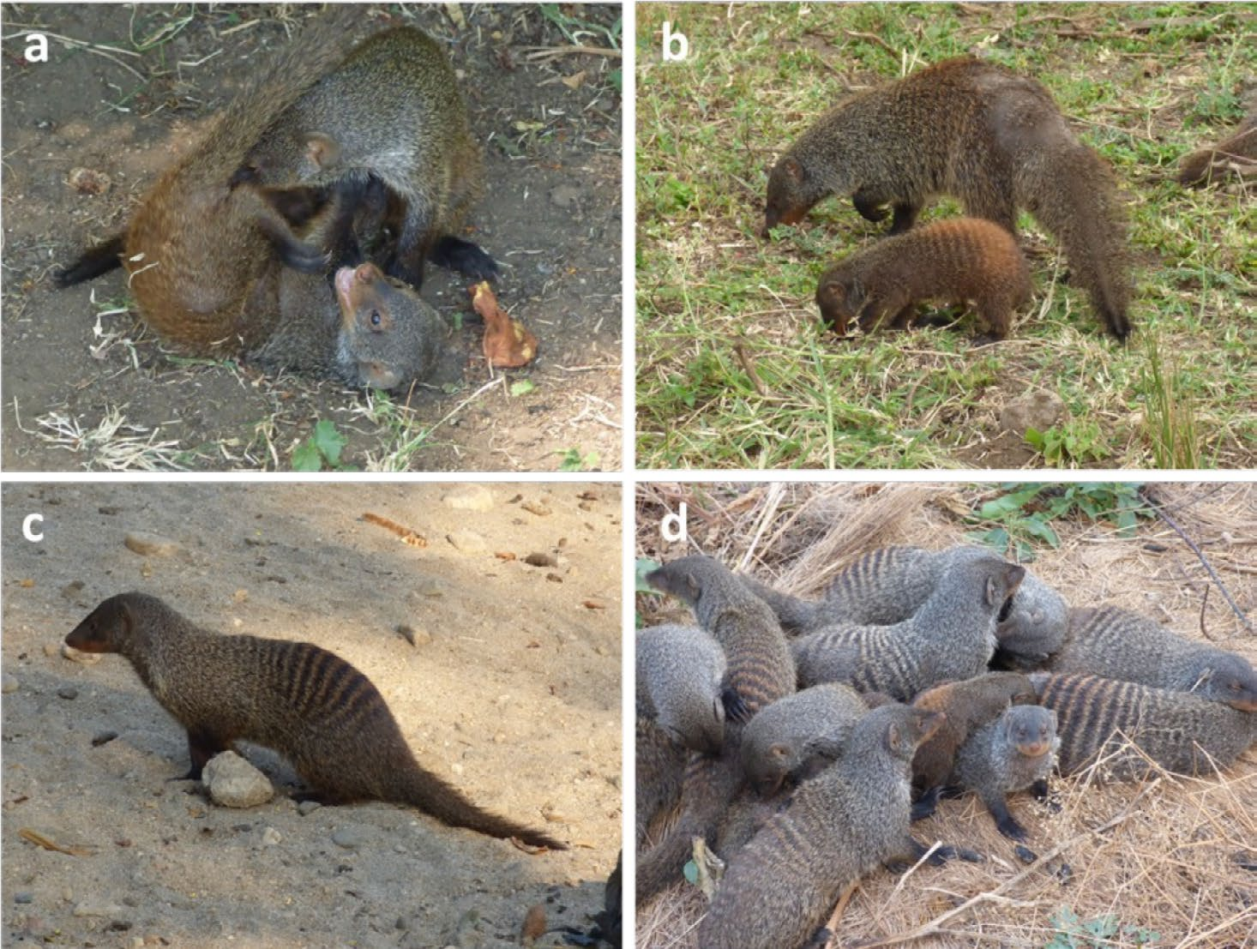
Common banded mongoose behaviors include (a) active socialization, (b) foraging, (c) resting and (d) huddling. Photos by Dr. Hazel Nichols.

Using a handheld digital camcorder (model B0C249LWG, YinFun), we took 187 focal observations between 7:47 and 19:01 with a mean (± SD) of 16.7 focals per hour (±9.2) between 8:00–19:00, which represents approximately 93% of the 12 h of daylight during which banded mongooses are active and includes their peak activity hours. Each individual was observed during 1–8 focal sessions though most (33 of 38) were observed between 4 and 6 times. Focals were conducted in a randomised order, and we attempted to focus each individual the same number of times, but this was not always possible due to some individuals remaining at the den to babysit and the group sometimes splitting during foraging, leading to some individuals being unavailable for observation at a given time. Throughout each focal, we continuously observed the individual's behaviour for a mean (±SD) of 5.0 min (±0.6), with some variation due to individuals moving out of sight or to areas that we were unable to follow due to safety considerations (ranging from 2 min 21 s to 6 min 47 s). We initially attempted to record 203 focal observations, but in 16 cases, individuals moved out of sight early on in the observation session (in under 2 min), and so these observations were not included in our final dataset of 187 observations. We used BORIS software (V.8.19.42023‐05‐29) (Friard and Gamba 2016) to code the behaviours from the videos using the ethogram (Table 1, Figure 1) and to calculate the proportion of the focal period the individual spent performing each behaviour. Time spent out of sight was excluded from our observations and downstream calculations. During each focal, we took a humidity (%) and *T*_a_ (°C) reading, using a digital thermometer and humidity monitor (Pitasha, accuracy 1°C for ambient temperatures ranging from −50°C to 70°C and 5% for relative humidity ranging from 10% to 99%). The monitor was placed in the shade at ground level (i.e., at the same level as the mongooses) within 5 m of the focal mongoose.

**TABLE 1:** Ethogram describing the behaviors performed by banded mongooses ( *Mungos mungo* ).

Behaviour type	Behaviour	Description
Active	Walking	The individual uses all four limbs to propel itself forward. The hind limbs are used to produce most of the propulsive thrust whereas the forelimbs are used as struts (Taylor 1970). The head is pointed in the direction of movement.
	Running	The individual uses all four limbs to propel itself forward. There are no more than two feet on the ground at any given time point (Taylor 1970). The head is pointed in the direction of movement.
	Foraging	The individual has its nose to the ground and can be moving or stationary. It can also be eating food or using claws to dig.
	Active socialisation	The individual is interacting with another individual by running towards or away from it, jumping on it or biting it. This is usually in the form of play fighting; however, occasionally these are serious forms of aggression.
Inactive	Resting	The individual is either lying down with its ventral surface in contact with the ground or sitting with its posterior and hind legs in contact with the ground.
	Huddling	The individual is resting whilst their torso is in contact with at least one other individual. There were at least two other individuals within 0.1 m of the focal individual.
	Panting	The individual has its mouth open and is displaying rapid shallow breathing.

### 2.3 Statistical Analyses

All statistical models were constructed using *lme4 1.1.35.5* (Bates et al. 2015) in R 4.4.2 (R Core Team 2024). Models were constructed based on our knowledge of the system and our predictions of what might influence the response variables. We fitted a single global model for each response variable, chosen a priori to represent predictions based on our knowledge of the biology of our study system, and there was no model selection procedure to alter the explanatory variables post hoc. We have detailed the structure of the potential relationships between our variables in Figure S1 following Franks et al. (2025), which we used to determine the most appropriate models to run. We employed standard model checks following Crawley (2015), including visual checks for normality of residuals, heteroskedasticity, and outliers, and corrected for these where necessary (details of any corrections are given in the descriptions of each model). *p*‐values for each variable were obtained by removing the variable in question from the full model and performing a likelihood ratio test.

To investigate associations between ambient temperature and behaviour, we constructed a series of four generalised linear models (GLMs) with a binomial error structure. For these models, the proportion of the focal period spent on one of the following four behavioural categories was fitted as the response variable: (1) active (combined data for walking, running, actively socialising with other individuals and foraging), (2) resting, (3) foraging and (4) huddling. Environmental variables, *T*_a_ (°C) and humidity (%), along with time of day, were fitted as explanatory variables in each model after being mean‐centred and scaled as they were previously on very different scales. Time of day was included as a fourth power explanatory term to allow for a bimodal activity pattern with two foraging sessions per day (Cant et al. 2013). This is also a potential ‘confounder’ variable, as it impacts both ambient temperature and likely has independent effects on behaviour, and therefore needs to be controlled for (see Data S1). As previous studies have found age and sex dependent effects of environmental conditions on banded mongooses (Marshall et al. 2016), we included the sex and age of the focal individual in the models to control for potential variation related to those attributes. We did not include the identity of the focal mongoose as a random effect, as preliminary mixed models estimated the variance of this random effect as zero, and including it resulted in a singular fit. We found collinearity between humidity and *T*_a_ (Pearson's correlation; *r* = −0.768) likely because air can hold more water at higher temperatures leading to a decrease in % humidity at a given absolute amount of water vapour in the air. Furthermore, humidity is likely to impact thermoregulatory behaviour through its effect on evaporative cooling (Powers 1992; Gerson et al. 2014; Mitchell et al. 2018). *T*_a_ can therefore have direct impacts on behaviour and also indirect impacts through its effect on humidity (see Data S1). As we were largely interested in the total impact of temperature on humidity (both direct and indirect), we excluded humidity from our model following (Franks et al. 2025). However, we also included a supplementary analysis incorporating humidity to explore the direct and indirect effects of temperature on behaviour (see Analysis S1).

Thermoregulation could also be influenced by the temperature gradient between the surface of the body (T_bs_) and *T*_a_ (Terrien et al. 2011). We therefore took T_bs_ recordings using a handheld visual infrared thermometer (see Analysis S2) and repeated our four models with the difference between average T_bs_ and *T*_a_ as an explanatory variable, instead of *T*_a_. However, all of these models (with the exception of huddling) had higher Akaike information criterion (corrected for sample size; AICc) values (Johnson and Omland 2004) than models with *T*_a_ (ΔAICc ranging from 0.55 to 9.43; Table S2), indicating that using *T*_a_ results in better models than using the temperature gradient between the T_bs_ and the environment. For consistency, we present the huddling model with *T*_a_ in the main text as there was no qualitative difference in the results when using the temperature gradient (Table S3). The better performance of models with *T*_a_ compared to the temperature gradient may be because T_bs_ increases linearly with *T*_a_ (Analysis S3, Table S4, Figure S2).

Whilst the main focus of our study was the association between *T*_a_ and behaviour (in the context of understanding the potential impacts of anthropogenic climate change), solar radiation is also known to have a strong impact on thermoregulation (Mitchell et al. 2018). In our study, the amount of solar radiation that a focal mongoose was exposed to could change within seconds, depending on cloud cover and vegetation cover and type, and we did not have ethical clearance from the Ugandan authorities to attach light‐monitoring tags to our study animals. It was therefore not feasible to collect detailed measurements representing the sun exposure of our focal mongooses. Instead, to give a broad indication of the impact of solar radiation on behaviour, we compared models without sunlight data to models including three categories of potential sunlight exposure: fully clouded over (0), partly cloudy (1) or clear sky (2). However, these categories of potential sunlight exposure had little detectable impact on behaviours performed, which may suggest that our broad categories of sun exposure were not reflective of the actual exposure of our focal mongoose to solar radiation. As the inclusion of sun exposure did not qualitatively change our results, they were excluded from the full model (Analysis S4, Table S5). Finally, to test the possibility of a non‐linear association between ambient temperature and behaviours performed, we tested for a quadratic effect; however, this was not significant and so was not included in our final models.

### 2.4 Ethics

This study was approved by the Ethical Review Committees of the Universities of Exeter and Swansea (010323/4401), the Uganda Wildlife Authority (COD/96/05) and Uganda National Council for Science and Technology (NS443ES) and adhered to the ‘Guidelines for the ethical treatment of nonhuman animals in behavioural research and teaching’, published by ASAB Ethical Committee/ABS Animal Care Committee (2025).

## 3 Results

Temperature and humidity varied substantially over the course of our study, with *T*_a_ ranging from 25.3°C to 37.2°C. This temperature range is reflective of the normal range of temperatures to which this study population is exposed: of the 7841 records of daily maximum temperatures that we have from our field site, 94.4% are within the range of temperatures that we recorded during our current study (Figure S3). Over the course of our study, humidity ranged from 45% to 79%. Temperatures were usually higher in the middle of the day, but there was considerable independent variation between these two variables (Pearson's correlation; *r* = 0.592), allowing the model to detangle their effects on behaviour (Morrissey and Ruxton 2018) (Figure S4). The most frequently performed behaviour was foraging (mean ± SE 54% ± 2.8% of the time budget), followed by resting (21% ± 2.2%), huddling (11% ± 2.0%), walking (9% ± 0.9%), active social behaviour (0.7% ± 0.3%) and running (0.5% ± 0.2%). Panting was not observed during the course of our data collection.

**FIGURE 2:**
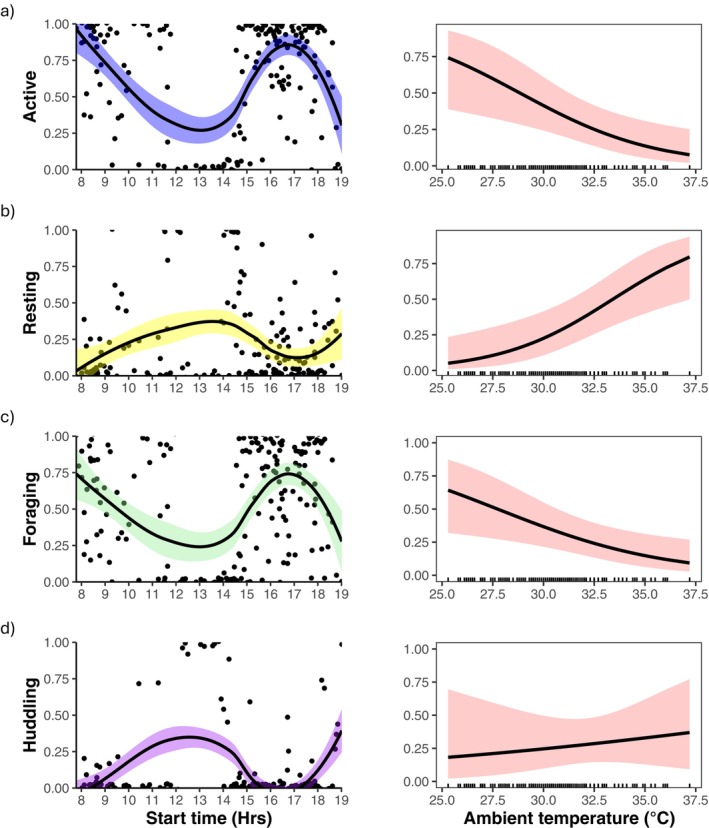
The proportion of time banded mongooses spent (a) active, (b) resting, (c) foraging and (d) huddling as a function of the time of day (left‐hand panels) and ambient shade temperature (*T*_a_) (°C) (right‐hand panels). Lines on the time plots (left‐hand panels) use a loess smoothing method, whilst lines on the right use predicted lines based on the general linear models (GLM). The shaded areas on all plots show the 95% confidence intervals. Model predictions for the effect of *T*_a_ (°C) on each behaviour (right‐hand panels) was obtained using the ‘ggpredict’ function from ggeffects 2.0.0 (Lüdecke 2018) and plotted using ggplot2 3.5.1 (Wickham 2016).

All of the behaviours we investigated, except resting, varied non‐linearly depending on the time of day. Individuals were more likely to be active and foraging during the morning and afternoon but were more likely to be huddling in the middle of the day and in the evening (Table 2; Figure 2a,c,d). After accounting for these daily behavioural changes, banded mongooses were significantly less active, foraged less and rested more under higher *T*_a_s (Table 2; Figure 2a–c). In contrast, the proportion of time spent huddling did not vary with *T*_a_ (Table 2; Figure 2d). Humidity had no significant effect on the behaviours measured (Table S1), and including humidity in the model as a mediator variable had little impact on the effect of *T*_a_ on behaviour, suggesting that *T*_a_ largely influences behaviour directly, rather than through an indirect impact via humidity. Finally, whilst we found no behavioural differences between males and females, we did find that older individuals were more likely to rest than younger individuals (Table 2).

**TABLE 2:** Summary of four GLMs investigating activity level and the proportion of time spent performing three behaviours: Resting, foraging and huddling.

Response	Fixed effects	Estimate	SE	Deviance	*p*
Active	(Intercept)	−0.179	0.423		
	**Ambient temperature**	**−0.707**	**0.257**	**8.686**	**3.21 × 10** ^**−3**^
	Sex (Male)	−0.299	0.392	0.588	0.443
	Age	−0.303	0.178	2.897	0.089
	**Start time**	**3.016**	**0.583**	**35.364**	**3.91 × 10** ^**−7**^
	**Start time^2**	**2.665**	**0.726**		
	**Start time^3**	**−2.137**	**0.413**		
	**Start time^4**	**−1.388**	**0.328**		
Resting	(Intercept)	−1.459	0.489		
	**Ambient temperature**	**0.854**	**0.278**	**10.841**	**9.93 × 10** ^**−4**^
	Sex (Male)	0.381	0.450	0.735	0.391
	**Age**	**0.419**	**0.181**	**5.254**	**0.022**
	Start time	−1.531	0.618	6.700	0.153
	Start time^2	−1.182	0.798		
	Start time^3	1.076	0.447		
	Start time^4	0.690	0.362		
Foraging	(Intercept)	−0.438	0.397		
	Ambient temperature	**−0.569**	**0.224**	**7.094**	**7.74 × 10** ^**−3**^
	Sex (Male)	−0.212	0.349	0.370	0.543
	Age	−0.242	0.168	2.128	0.145
	**Start time**	**2.351**	**0.541**	**22.989**	**1.27 × 10** ^**−4**^
	**Start time^2**	**1.856**	**0.657**		
	**Start time^3**	**−1.588**	**0.381**		
	**Start time^4**	**−1.008**	**0.294**		
Huddling	(Intercept)	−0.920	0.583		
	Ambient temperature	0.192	0.375	0.261	0.610
	Sex (Male)	−0.165	0.572	0.083	0.774
	Age	−0.212	0.289	0.560	0.454
	**Start time**	**−4.697**	**1.078**	**36.145**	**2.70 × 10** ^**−7**^
	**Start time^2**	**−4.339**	**1.310**		
	**Start time^3**	**3.271**	**0.722**		
	**Start time^4**	**2.120**	**0.589**		

*Note:* Our model included data from 187 focal recordings of 38 banded mongooses. The table shows the estimates and associated standard errors (SE) from the models, along with the deviance and *p*‐value associated with removing the term from the model. Significant terms (*p* < 0.05) are shown in bold.

## 4 Discussion

We found that banded mongooses appear to modify their behaviour to thermoregulate in response to high ambient temperatures (*T*_a_s) by reducing activity levels and increasing time spent resting. Only 11% (27/245) of resting bouts occurred in the sun, but this was never during the middle of the day (12:00–15:00) (0/27). Our results support the thermal constraint hypothesis, which suggests that for animals seeking to maximise their food gain, foraging is constrained by their thermal tolerance (Cerdá et al. 1998; Pereboom and Biesmeijer 2003; Spicer et al. 2017). Similar results have been found in Azara's owl monkeys ( *Aotus azarae* ) whereby resting increased whilst foraging reduced as *T*_a_s increased (Perea‐Rodríguez et al. 2022), and also in tropical ant species, where workers stopped foraging under high *T*_a_s but resumed when *T*_a_s cooled down (Spicer et al. 2017). These behavioural modifications occurred in banded mongooses in response to changes in ambient temperature even after accounting for the time of day, suggesting that other changes in temperature (not related to time of day), such as day‐to‐day variation, also affect behaviour.

We measured behaviour under a relatively ‘typical’ range of temperatures for banded mongooses in Uganda. The TNZ is most likely around 30.7°C–33.4°C (Sadie 1983) for resting banded mongooses in a laboratory setting. However, this has limited applicability to mongooses in the wild, where activity levels are much higher, so preferred temperatures may be lower. In our study, the maximum temperature recorded was 37.2°C and 9.6% of our focal observations were at temperatures above 33.4°C. Furthermore, our study focused on behavioural thermoregulation, which is likely to occur before animals start to experience negative physiological impacts due to rises in *T*_a_. Future studies would benefit from further investigating the impact of thermal extremes on banded mongoose fitness, for example, by measuring behaviour during heat waves. This may give a better idea of how they might respond to increased thermal extremes due to future climate change. Nevertheless, our study provides an excellent basis for understanding how banded mongoose behaviour changes over the range of temperatures that they are typically exposed to.

Thermal constraints on foraging likely have direct fitness consequences, resulting in a decrease in energy intake which can lead to reduced energy allocations to growth, maintenance and reproduction (Gadgil and Bossert 1970; Kramer and Ellison 2010). For example, once banded mongoose pups have been weaned and accompany the group on foraging trips, adult banded mongooses are less likely to engage in pup care under hot conditions, presumably to prioritise their own survival, leading to high pup mortality (Khera 2024). Similar patterns have been found in African wild dogs ( *Lycaon pictus* ), whereby the foraging time is reduced under hot conditions (Woodroffe et al. 2017) and adults struggling to meet food intake requirements are thought to prioritise foraging over helping, leading to higher pup mortality (Courchamp et al. 2002). The indirect impacts of high *T*_a_s on offspring mortality, which arise due to behavioural changes in adults, may therefore have substantial impacts on the viability of some cooperatively breeding animal populations under future climate change scenarios (Rabaiotti et al. 2023).

The cost of reduced time spent foraging is also likely to depend on food availability. As our study took place towards the end of the wet season where natural food availability was likely to be relatively high (Rood 1975), banded mongooses may have been able to reduce foraging time without reducing food intake beyond what is needed to maintain their body condition. During the dry season, however, when food is more scarce, banded mongooses may face a stronger trade‐off between foraging and thermoregulation. Supporting this, lower food availability has been found to increase the amount of time spent foraging occurring under high temperatures in hoopoe‐larks ( *Alaemon alaudipes* ) (Tieleman and Williams 2002). Future studies could address this by investigating how temperature impacts banded mongoose behaviour and body mass over a longer time period covering both wet and dry seasons.

Foraging during cooler environmental conditions and resting when conditions are hot is a common strategy employed by species living in hot climates and often results in a bimodal activity pattern with individuals being more active and foraging during the morning and late afternoon and resting during the middle of the day when *T*_a_s are highest. We found this foraging pattern in banded mongooses, with individuals being more active and foraging between 8:00–10:00 and 15:00–18:00 and resting in the shade between 12:00 and 15:00 (pers. obs.). A lack of foraging during the middle of the day may be facilitated by satiation after the morning foraging session. However, we find impacts of temperature, after accounting for time, suggesting that temperature likely impacts foraging directly. Similar patterns have been found in other species including Iberian rabbits ( *Oryctolagus cuniculus algirus* ) (Rocha et al. 2022), wall lizards ( *Podarcis muralis* ), Azara's owl monkeys (Perea‐Rodríguez et al. 2022) and multiple desert bird species (Goldstein 1984; Carmi‐Winkler et al. 1987; Hinsley 1994; Williams 2001). In more extreme cases, high daytime *T*_a_s can lead to temporal niche switching, whereby normally diurnal species may switch to foraging at night to avoid high *T*_a_s (Hunt Jr. 1977; Maloney et al. 2005; Davimes et al. 2017; Brivio et al. 2024). For example, temporal niche switching is shown in the Arabian oryx (*Octocyon megalotis*), which is diurnal during the winter months but becomes nocturnal or crepuscular during the hot summer months when maximum *T*_a_s can reach highs of 45°C (Davimes et al. 2017). Banded mongooses, however, are strictly diurnal (Rood 1975, pers. obs.) and so do not use a temporal niche switching strategy to avoid high daytime *T*_a_s possibly due to high predation risk at night.

Huddling has previously been suggested as a form of thermoregulation for banded mongooses, though in response to cool rather than hot conditions (Rood 1975), such that we expected to find less huddling in hot conditions. However, in contrast to our predictions, we found no effect of *T*_a_ on the proportion of time spent huddling, which is unexpected given that huddling is strongly linked to thermoregulation in other species where this behaviour occurs (Donati et al. 2011; Eppley et al. 2017). Although our study was not designed to evaluate other potential explanations for huddling, this behaviour was more common immediately after foraging sessions. This could suggest that huddling is a method of re‐establishing social bonds following greater dispersion during foraging sessions. This hypothesis has seldom been explored in studies investigating huddling behaviour, but Kelley et al. (2016) and Gestich et al. (2014) also found a lack of association between huddling and *T*_a_s in ring‐tailed lemurs ( *Lemur catta* ) and black‐fronted titi monkeys ( *Callicebus nigrifrons* ) respectively, and proposed similar social explanations for the behaviour.

## 5 Conclusions

We find that banded mongooses appear to behaviourally thermoregulate by foraging less and resting more when ambient temperatures (*T*_a_s) are high, which could explain the bimodal activity pattern observed. However, even after accounting for changes in behaviour related to the time of day, we find that ambient temperature is associated with behavioural patterns, which suggests that temperature changes impact behaviour over and above their association with the time of day. Changes in foraging behaviour may also explain why high *T*_a_s have previously been shown to reduce pup survival, pup weight and the pup‐care behaviour of adults (Khera 2024). It is therefore possible that banded mongooses may struggle to cope with future rises in *T*_a_s resulting from anthropogenic climate change. Our results demonstrate that behavioural thermoregulation may result in trade‐offs between thermoregulation and foraging that could reduce fitness, potentially leading to population declines under climate change, even for equatorial species that are adapted to relatively high *T*_a_s. Future studies on banded mongooses would benefit from investigating the impacts of extreme temperatures (such as heat waves) on foraging behaviour and fitness and investigating whether these impacts may vary in the dry versus wet seasons. Furthermore, addressing the impacts of changing environmental conditions on a wider range of equatorial species would help us to understand the broader impacts, and where feasible, studies should use tags attached directly to the animals, which can measure environmental conditions (via data loggers) and record behavioural data (via an accelerometer). Such studies could reveal much finer scale details of thermoregulatory behaviour, thus providing further information on how equatorial animals are likely to respond to climate change.

## Author Contributions

**Monil Khera:** conceptualization (equal), formal analysis (lead), funding acquisition (equal), investigation (equal), methodology (equal), project administration (equal), validation (equal), visualization (lead), writing – original draft (lead), writing – review and editing (equal). **Kevin Arbuckle:** conceptualization (equal), investigation (equal), methodology (equal), supervision (supporting), writing – review and editing (equal). **Onismus Bwambale:** investigation (equal), writing – review and editing (equal). **Francis Mwanguhya:** funding acquisition (equal), project administration (equal), validation (equal), writing – review and editing (equal). **Megan H. Nicholl:** formal analysis (supporting), writing – review and editing (equal). **Michael A. Cant:** funding acquisition (equal), project administration (equal), validation (equal), writing – review and editing (equal). **Hazel J. Nichols:** conceptualization (equal), funding acquisition (equal), investigation (equal), methodology (equal), project administration (equal), supervision (lead), validation (equal), writing – review and editing (equal).

## Conflicts of Interest

The authors declare no conflicts of interest.

## Supporting information


Data S1.

Figure S1.

Figure S2.

Figure S3.

Figure S4.

## Data Availability

The dataset and Rcode used in our analysis is available on Dryad: https://doi.org/10.5061/dryad.866t1g23j.
