# Supplementary material for: High Ambient Temperatures Are Associated With Reduced Foraging Capacity in an Equatorial Mammal, the Banded Mongoose (Mungos mungo)
Source: Ecol Evol. 2025 Jul 29;15(8):e71872. doi: 10.1002/ece3.71872 (PMC12305352; doi:10.1002/ece3.71872)
Supplement: Supplementary file 1 — Data S1. [file ECE3-15-e71872-s003.doc]

**Supplementary material: High ambient temperatures constrain foraging capacity in an equatorial mammal; the banded mongoose**

**Supplementary diagram. Justification of model choice along with a diagram of potential causal pathways.**

The primary aim of our study was to investigate the impact of ambient temperature on behaviour. Ambient temperature could either directly or indirectly affect behaviour (through its effect on humidity) since higher ambient temperatures increase the amount of water vapour that can be held in the air, leading to a decrease in relative humidity (%) for a given absolute amount of water vapor in the air. This explains the negative relationship observed between humidity (%) and ambient temperature (see supplementary figure 2). Humidity in turn impacts on evaporative cooling, with lower levels of humidity facilitating evaporative cooling (as detailed in our introduction). Humidity is therefore a ‘mediator’ variable between temperature and behaviour (Frank *et al*. 2025). Mediators are essential for distinguishing between the total causal effect (represented by all green pathways in Fig.S1) and the direct causal effect of temperature on behaviour (represented by the arrow directly between these two variables). As we were primarily interested in the total causal effect of temperature on behaviour, we excluded humidity from the main model (see Franks *et al*. 2025).

The start time of our focal observation was a potential ‘confounder’ variable as it impacts the air temperature and is also likely to have independent impacts on behaviour (see Fig.S1). We therefore included this variable in our model to control for it in order to prevent potentially misleading conclusions regarding the impact of ambient temperature on behaviour (Franks *et al*. 2025). We also included sex and age in our model as these are ‘competing exposure’ variables which could explain some of the variance in behaviour and therefore the inclusion of these variables may help to provide a more accurate assessment of the impact of temperature on behaviour.

Figure S1. Directed acyclic graph of the proposed causal pathways in our dataset, created using DAGitty web app (Textor *et al*. 2016), following Franks *et al*. (2025). Green arrows represent potential causal pathways between our two variables of interest (air temperature and behaviour). Red pathways indicate ‘confounder variables’, which could influence both variables of interest, and black pathways represent ‘competing exposure’ variables, which impact only our response variable (behaviour).

**Supplementary analysis 1. Testing the robustness of our behaviour models by including humidity**

Table S1. Summary of four GLMs investigating activity level and the proportion of time spent performing three behaviours: resting, foraging, and huddling, with humidity included as an additional explanatory variable. Our model included data from 187 focal recordings of 38 banded mongooses. The table shows the estimates and associated standard errors (SE) from the models.

| **Response** | **Fixed effects** | **Estimate** | **SE** | **Deviance** | **P-value** |
| --- | --- | --- | --- | --- | --- |
| Activity | (Intercept) | -0.279 | 0.438 |  |  |
|  | **Ambient temperature** | **-0.945** | **0.326** | **9.486** | **2.07x10^-3^** |
|  | Sex (Male) | -0.355 | 0.398 | 0.811 | 0.368 |
|  | Age | -0.324 | 0.179 | 3.278 | 0.070 |
|  | Humidity | -0.375 | 0.302 | 1.538 | 0.215 |
|  | **Start time** | **3.013** | **0.580** | **36.494** | **2.29x10^-7^** |
|  | **Start time^2** | **2.921** | **0.765** |
|  | **Start time^3** | **-2.149** | **0.412** |
|  | **Start time^4** | **-1.452** | **0.335** |
| Resting | (Intercept) | -1.386 | 0.492 |  |  |
|  | **Ambient temperature** | **1.071** | **0.354** | **10.395** | **1.26x10^-3^** |
|  | Sex (Male) | 0.432 | 0.456 | 0.927 | 0.336 |
|  | **Age** | **0.442** | **0.182** | **5.747** | **0.017** |
|  | Humidity | 0.364 | 0.351 | 1.078 | 0.299 |
|  | Start time | -1.516 | 0.618 | 6.880 | 0.142 |
|  | Start time^2 | -1.365 | 0.820 |
|  | Start time^3 | 1.073 | 0.446 |
|  | Start time^4 | 0.725 | 0.365 |
| Foraging | (Intercept) | -0.567 | 0.411 |  |  |
|  | **Ambient temperature** | **-0.861** | **0.297** | **9.338** | **2.24x10^-3^** |
|  | Sex (Male) | -0.263 | 0.354 | 0.557 | 0.455 |
|  | Age | -0.263 | 0.168 | 2.486 | 0.115 |
|  | Humidity | -0.429 | 0.272 | 2.513 | 0.113 |
|  | **Start time** | **2.369** | **0.540** | **24.162** | **7.411x10^-5^** |
|  | **Start time^2** | **2.182** | **0.701** |
|  | **Start time^3** | **-1.619** | **0.382** |
|  | **Start time^4** | **-1.100** | **0.303** |
| Huddling | (Intercept) | -0.916 | 0.590 |  |  |
|  | Ambient temperature | 0.203 | 0.445 | -0.210 | 0.647 |
|  | Sex (Male) | -0.162 | 0.577 | -0.078 | 0.780 |
|  | Age | -0.210 | 0.290 | -0.550 | 0.459 |
|  | Humidity | 0.024 | 0.508 | -0.002 | 0.962 |
|  | **Start time** | **-4.698** | **1.079** | **-35.960** | **2.949x10^-7^** |
|  | **Start time^2** | **-4.346** | **1.317** |
|  | **Start time^3** | **3.270** | **0.723** |
|  | **Start time^4** | **2.119** | **0.589** |

**Supplementary analysis 2. Comparing models including T_a_ to those including the difference between T_a_ and T_bs_**

**Measuring body surface temperature**

Body surface temperature (T_bs_) is likely to influence thermoregulation through impacting the temperature gradient between the surface of the body and T_a_ (Terrien *et al*. 2011). During each focal, we took a mean of 5 (range 1–14) T_bs_ recordings using a handheld visual infrared thermometer (Model IRO280H, Perfect Prime, accuracy 0.3°C within mammalian body temperature range 32°C - 42°C) from a distance of ~50cm (as recommended by the manufacturer) and emissivity set to 0.98, which is representative of animal body surfaces (Mitchell et al., 2018). Average temperature readings were taken from the head incorporating the eye and ear region, as these areas have little hair and so best represent T_bs_ (McCafferty et al. 2011) and readings were excluded from the analysis if they incorporated areas that were not part of the animal’s surface. We also avoided taking temperatures from the nose area as, whilst lacking hair, the nose was often placed in damp or cool areas during foraging, which would likely result in unrepresentative skin temperatures. We used the mean T_bs_ (°C) reading per focal in our analyses.

Table S2. Model comparison table created using windex 2.0.8 (Arbuckle & Minter 2015) showing competing models from our analyses where both models include the following fixed effects: sex, age, and start time as a fourth power term. One model however included ambient temperature (T_a_) as a fixed effect (M1) whilst the other included the difference between body surface temperature (T_bs_) and T_a_ instead (M2). The table includes the name of the model, the number of parameters (K), logLik (log-likelihood), AIC, deltaAICc (the difference between the best model and every other model), Weight (model probabilities) and Evidence ratio (the amount of evidence for the best model relative to each model i.e.. a score of 3 means that there is 3 times less evidence supporting it than the best model).

| **Response variable** | **Model** | **K** | **logLik** | **AICc** | **deltaAICc** | **Weight** | **Evidence.ratio** |
| --- | --- | --- | --- | --- | --- | --- | --- |
|  | M1 | 8 | -78.12 | 173.04 | 0.00 | 0.99 | 1.00 |
| Activity | (Ambient temperature) |  |  |  |  |  |  |
|  | M2 | 8 | -82.83 | 182.47 | 9.43 | 0.01 | 111.72 |
|  | (Difference in temperature) |  |  |  |  |  |  |
|  | M1 | 8 | -57.77 | 132.35 | 0.00 | 0.57 | 1.00 |
| Resting | (Ambient temperature) |  |  |  |  |  |  |
|  | M2 | 8 | -58.04 | 132.90 | 0.55 | 0.43 | 1.32 |
|  | (Difference in temperature) |  |  |  |  |  |  |
|  | M1 | 8 | -101.24 | 219.28 | 0.00 | 0.97 | 1.00 |
| Foraging | (Ambient temperature) |  |  |  |  |  |  |
|  | M2 | 8 | -104.79 | 226.39 | 7.11 | 0.03 | 34.93 |
|  | (Difference in temperature) |  |  |  |  |  |  |
|  | M2 | 8 | -43.55 | 103.91 | 0.00 | 0.58 | 1.00 |
| Huddling | (Difference in temperature) |  |  |  |  |  |  |
|  | M1 | 8 | -43.88 | 104.56 | 0.66 | 0.42 | 1.39 |
|  | (Ambient temperature) |  |  |  |  |  |  |

Table S3. Summary of a GLM investigating the proportion of time spent huddling. Our model included data from 187 focal recordings of 38 banded mongooses. The table shows the estimates and associated standard errors (SE) from the models, along with the deviance and P-value associated with removing the term from the model and performing a likelihood ratio test. Significant terms (P<0.05) are shown in bold.

| **Fixed effects** | **Estimate** | **SE** | **Deviance** | **P-value** |
| --- | --- | --- | --- | --- |
| (Intercept) | -0.82558 | 0.55053 |  |  |
| Difference in temperature | 0.42047 | 0.32604 | 1.7633 | 0.1842193 |
| Sex (M) | -0.09873 | 0.57534 | 0.0293 | 0.8640651 |
| Age | -0.19837 | 0.29938 | 0.4557 | 0.4996392 |
| **Start time** | **-4.45904** | **1.03323** |  |  |
| **Start time^2** | **-4.14986** | **1.32626** | 36.48 | **2.305x10^-7^** |
| **Start time^3** | **3.02916** | 0.68674 |  |  |
| **Start time^4** | **1.89434** | 0.58565 |  |  |

**Supplementary analysis 3. Investigating how T_bs_ varies with T_a_ and humidity**

We also investigated how banded mongoose body surface temperature (T_bs_) varies with ambient temperature (T_a_). T_bs_s are tightly associated with an animal’s microclimate (McFarland *et al*. 2020), so a dissociation between T_bs_ and T_a_ could indicate that banded mongooses minimise heat gain from the environment by avoiding microclimates with high T_a_s. To investigate the impact of changing T_a_ and humidity on T_bs_, we constructed a linear mixed effects model (LMM) with mean T_bs_ during the focal as the response variable and scaled environmental conditions (T_a_ and humidity) along with scaled age and sex fitted as explanatory variables. The identity of the individual focalled was also included as a random effect. P-values for each variable were obtained by removing the variable in question from the full model and performing a likelihood ratio test. T_bs_ significantly increased as T_a_ rose, but significantly decreased as humidity increased (Table S4; Fig. S2). The relationship between T_a_ and T_bs_ was linear (there was no significant improvement in the model when adding T_a_ as a quadratic term). Model predictions for the effect of T_a_ and humidity on T_bs_ were obtained using the ‘ggpredict’ function from *ggeffects 2.0.0* (Lüdecke, 2018) and plotted using *ggplot2 3.5.1* (Wickham, 2016).

| **Fixed effects** | **Estimate** | **SE** | **χ^2^** | **P-value** |
| --- | --- | --- | --- | --- |
| (Intercept) | 0.378 | 0.147 |  |  |
| **Ambient temperature** | **0.645** | **0.130** | **23.677** | **1.139 x10^-6^** |
| Sex (Male) | 0.055 | 0.182 | 0.098 | 0.754 |
| Age | -4.632x10^-4^ | 0.086 | 0.00 | 0.999 |
| **Humidity** | **-0.382** | **0.130** | **8.684** | **3.21x10^-3^** |

Table S4. Summary of a LMM investigating the effect of environmental conditions on average T_bs_ (°C) from 187 focal recordings of 38 banded mongooses. The table shows the estimates and associated standard errors (SE) from the models, along with the chi-squared value (χ^2^) and P-value associated with removing the term from the model and performing a likelihood ratio test.

Fig. S2. The effect of (a) ambient temperature (°C ) and (b) humidity on T_bs_). Lines show the predictions from the LMM and the shaded areas represent the 95%CI.

**Supplementary analysis 4. Comparing models with and without sunlight**

Table S5. Model comparison table using Chi-squared to determine the effect of sunlight on four GLMs investigating activity level and the proportion of time spent performing three behaviours: resting, foraging, and huddling. The table shows the residual degrees of freedom and residual deviance for both the full model (M1) and the model without sunlight (M2), along with the degrees of freedom, deviance, and P-value associated with removing sunlight from the full model and performing a likelihood ratio test.

| **Response variable** | **Model** | **Resid.df** | **Resid.dev** | **df** | **Deviance** | **P-value** |
| --- | --- | --- | --- | --- | --- | --- |
| Activity | M1  (with sunlight) | 176 | 87.173 | 2 | 0.159 | 0.924 |
|  | M2  (without sunlight) | 178 | 87.332 |  |  |  |
| Resting | M1  (with sunlight) | 176 | 68.649 | 2 | 2.2497 | 0.287 |
|  | M2  (without sunlight) | 178 | 71.146 |  |  |  |
| Foraging | M1  (with sunlight) | 176 | 99.124 | 2 | 0.118 | 0.943 |
|  | M2  (without sunlight) | 178 | 99.242 |  |  |  |
| Huddling | M1  (with sunlight) | 176 | 55.435 | 2 | 5.591 | 0.061 |
|  | M2  (without sunlight) | 178 | 61.026 |  |  |  |

**Supplementary plot 1. Plot of maximum T_a_s recorded at our study site compared to range of T_a_s collected during our study.**

Figure S3. A histogram of the maximum daily temperatures recorded at our field site. Blue dotted lines represent the minimum and maximum temperatures recorded during the current study. These bounds incorporate 96.8% of the daily temperature records at our field site.

**Supplementary plot 2. Plot of T_a_ at different times of day**

Fig. S4. Ambient shade temperatures (°C) at the start of each focal session over the course of the day.

**Reference list**

Arbuckle K, Minter, A., Windex: Analyzing convergent evolution using the Wheatsheaf index in R. Evolutionary Bioinformatics 2015; 11: EBO-S20968.

Franks DW, Ruxton GD, Sherratt T., Ecology needs a causal overhaul. Biological Reviews 2025. Online Early.

Lüdecke D., ggeffects: Tidy Data Frames of Marginal Effects from Regression Models, Journal of Open Source Software 2018; 3:772.

McCafferty DJ, Gilbert C., Paterson W, et al., Estimating metabolic heat loss in birds and mammals by combining infrared thermography with biophysical modelling, Comparative Biochemistry and Physiology Part A: Physiology 2011; 158: 337–345.

McFarland R., Barrett, L., Fuller, A., Hetem, R. S., Porter, W. P., Young, C., & Henzi, S. P. . Infrared thermography cannot be used to approximate core body temperature in wild primates. American Journal of Primatology 2020; 82: e23204.

Mitchell D, Snelling EP, Hetem RS, et al., Revisiting concepts of thermal physiology: Predicting responses of mammals to climate change, Journal of Animal Ecology 2018; 87: 956–973.

Terrien J, Perret M, Aujard F., Behavioural thermoregulation in mammals: a review, Frontiers in Bioscience 2011; 16: 1428–1444.

Textor, J., Van der Zander, B., Gilthorpe, M.S., Liśkiewicz, M. and Ellison, G.T., 2016. Robust causal inference using directed acyclic graphs: the R package ‘dagitty’. International journal of epidemiology, 45(6), pp.1887-1894.

Wickham H., ggplot2: Elegant Graphics for Data Analysis, 2016. Springer-Verlag New York.
