# Supplementary figures and images for: High Ambient Temperatures Are Associated With Reduced Foraging Capacity in an Equatorial Mammal, the Banded Mongoose (Mungos mungo)
Source: Ecol Evol. 2025 Jul 29;15(8):e71872. doi: 10.1002/ece3.71872 (PMC12305352; doi:10.1002/ece3.71872)

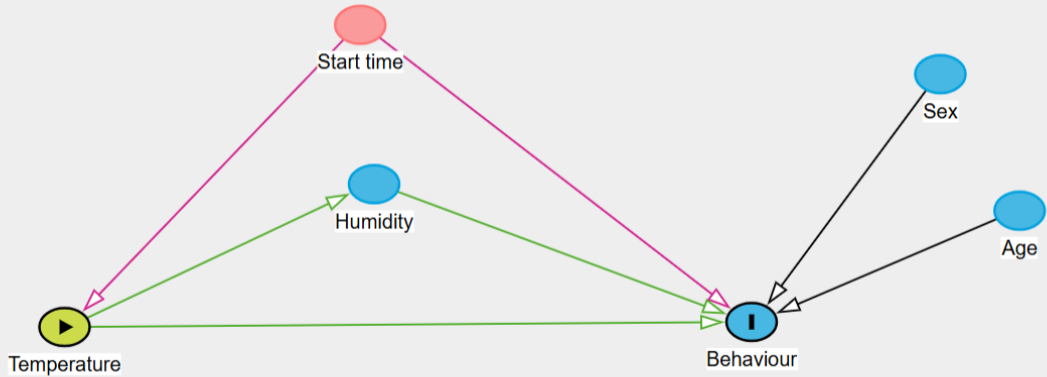

Supplement: Supplementary file 2 — Figure S1. [file ECE3-15-e71872-s002.pdf]

a)

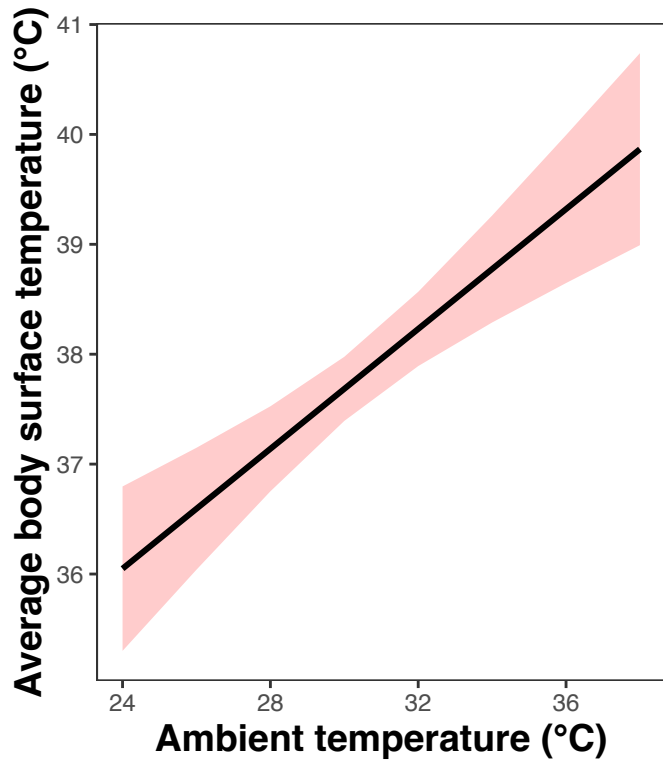

b)

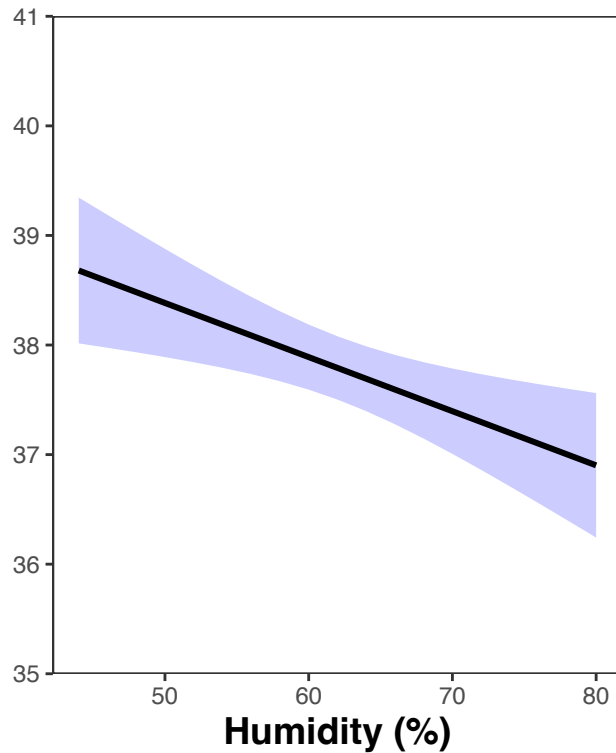

Supplement: Supplementary file 3 — Figure S2. [file ECE3-15-e71872-s004.pdf]

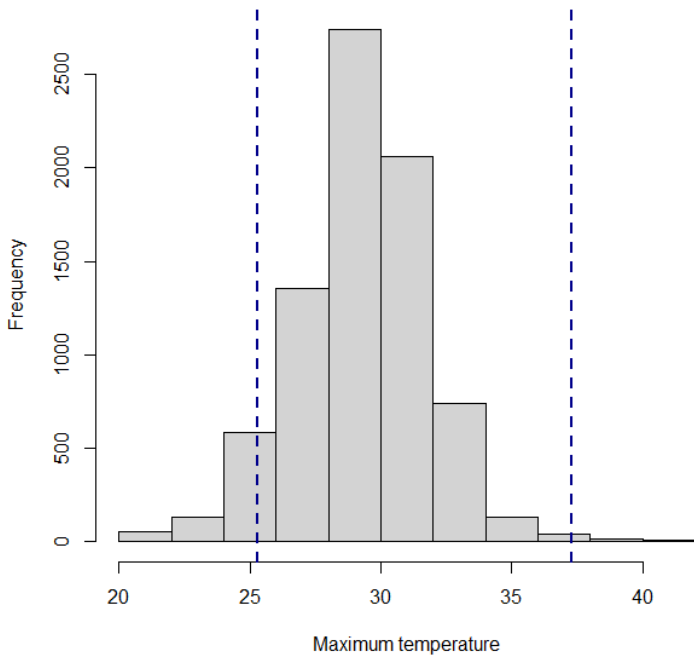

Supplement: Supplementary file 4 — Figure S3. [file ECE3-15-e71872-s005.pdf]

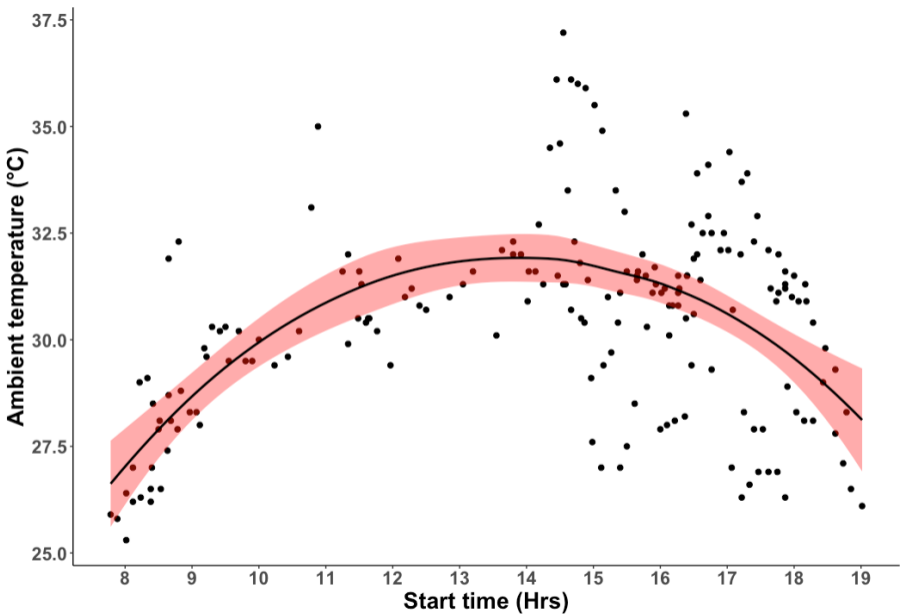

Supplement: Supplementary file 5 — Figure S4. [file ECE3-15-e71872-s001.pdf]
